# The psychological configuration of suicidal ideation in depressive disorder: an integrative and predictive approach

**DOI:** 10.3389/fpubh.2026.1958636

**Published:** 2026-10-09

**Authors:** Sebastian Mihai Armean, Codruţa Alina Popescu, Bogdan Florin Covaliu, Bogdana Adriana Năsui, Roxana Şipoş, Romulus Dan Nicoară

**Affiliations:** 1 Department of Pharmacology, Toxicology and Clinical Pharmacology, “Iuliu Haţieganu” University of Medicine and Pharmacy, Cluj-Napoca, Romania; 2 Department of Medical Education, “Iuliu Haţieganu” University of Medicine and Pharmacy, Cluj-Napoca, Romania; 3 Department of Community Medicine, “Iuliu Haţieganu” University of Medicine and Pharmacy, Cluj-Napoca, Romania; 4 Department of Neuroscience, Psychiatry and Pediatric Psychiatry, “Iuliu Haţieganu” University of Medicine and Pharmacy, Cluj-Napoca, Romania

**Keywords:** depressive disorder, emotion regulation difficulties, maladaptive coping, suicidal ideation, suicide risk

## Abstract

**Background:**

Suicidal ideation in depression is increasingly understood as a multidetermined phenomenon shaped by emotional distress, emotion regulation deficits, maladaptive coping, and contextual vulnerability. The present study examined the psychological correlates and predictors of suicidal ideation and tested the mediating roles of emotion regulation difficulties and quality of life in adults with depressive disorder.

**Methods:**

A cross-sectional study was conducted on 172 adults with depressive disorder. Participants completed standardized measures of suicidal ideation and suicidal behavior (C-SSRS), depressive severity (MADRS), emotional distress (DASS-21R), emotion regulation difficulties (DERS-SF), coping strategies (Cognitive Emotion Regulation Questionnaire [CERQ] and Strategic Approach to Coping Scale [SACS]), and quality of life (QOLI). Spearman correlations, hierarchical regression analyses, and mediation models with bootstrapping (5,000 samples) were performed.

**Results:**

Suicidal ideation was positively associated with depressive severity, anxiety, stress, several dimensions of emotion regulation difficulties, and maladaptive coping strategies, and negatively associated with quality of life. Hierarchical regression analyses identified depressive severity and quality of life as the most robust contributors in the first model, while maladaptive behavioral and cognitive coping strategies contributed additional explanatory value in the second model. Mediation analyses indicated that emotion regulation difficulties significantly mediated the association between depressive severity and suicidal ideation, and that a significant serial indirect effect also operated through reduced quality of life. In both models, the direct effect of depressive severity remained significant, indicating partial mediation.

**Conclusions:**

Suicidal ideation in depressive disorder is associated with a broader psychological configuration involving emotional distress, emotion regulation difficulties, maladaptive coping, and diminished quality of life. These findings support an integrative, multivariable approach to understanding suicidal ideation in depression and highlight clinically relevant psychological factors that may inform assessment and intervention.

## 1 Introduction

Suicidal ideation has become an increasingly important target of empirical investigation because it represents a clinically meaningful outcome in its own right, not merely a preliminary stage of suicidal behavior (1, 2). Although suicide prevention research has traditionally prioritized attempts and deaths, more recent work has emphasized that persistent, severe, or escalating suicidal thoughts are themselves associated with substantial functional burden, psychiatric severity, and later suicidal outcomes (2). From a public health perspective, this focus is justified by the continuing global burden of suicide, which remains one of the leading causes of death among young people worldwide (1).

At the same time, contemporary suicidology increasingly rejects the assumption that suicidal ideation is a uniform phenomenon, stressing instead its heterogeneity in intensity, duration, controllability, temporal course, and proximity to suicidal behavior (2, 3). This heterogeneity has direct methodological implications, because studies that collapse ideation into a single undifferentiated indicator risk obscuring clinically relevant variation in suicide risk (4). In this regard, the Columbia–Suicide Severity Rating Scale has particular value because it allows the differentiation of suicidal ideation from suicidal behavior while also capturing gradations of ideational severity that are highly relevant for research and clinical assessment (5).

Among the psychological variables associated with suicidality, depression remains one of the most robust and consistently replicated correlates of suicidal ideation across clinical and nonclinical populations (6, 7). Recent longitudinal and meta-analytic evidence suggests that depressive symptom burden is not only associated with current suicidal ideation, but may also shape its trajectory over time and its persistence during treatment (6, 7). At the same time, newer studies show that depression alone does not fully explain why some individuals report suicidal ideation whereas others with comparable depressive severity do not, indicating the need for broader multivariate models of risk (6, 8).

This shift away from single-predictor models has been strengthened by recent literature showing that suicidal ideation often emerges within broader constellations of psychological distress that include anxiety, stress reactivity, sleep disturbance, and impaired psychosocial functioning. Such findings are consistent with the view that suicidal thinking is best understood as a multidetermined phenomenon embedded in an interacting network of emotional, cognitive, and contextual vulnerabilities rather than as the direct outcome of depressive affect alone (9, 10). Consequently, current research increasingly favors integrative frameworks that examine how multiple risk dimensions combine within individuals to produce different forms and intensities of suicidal ideation (9, 10).

One construct that has gained substantial prominence in this newer literature is emotion regulation, particularly because difficulties in modulating affective experience appear to intensify the transition from distress to suicidal thinking (11, 12). A recent systematic review and three-level meta-analysis concluded that emotion regulation is reliably associated with suicidal thoughts and behaviors, especially in younger populations, supporting its role as a transdiagnostic vulnerability factor rather than a diagnosis-specific correlate (11). Complementing this evidence, intervention-focused reviews have begun to show that emotion-regulation-targeted approaches are increasingly considered promising during acute suicidal crises, which further underscores the theoretical and clinical centrality of this construct (12).

At a more specific level, maladaptive cognitive emotion regulation strategies such as rumination, intrusive brooding, catastrophizing, and emotional suppression have been repeatedly associated with higher suicidal ideation in recent studies (13, 14). In contrast, more adaptive regulation processes may mitigate suicidal thinking indirectly by improving perceived coping efficacy, emotional resilience, and interpersonal functioning (13, 15).

Quality of life has likewise become increasingly relevant in recent studies of suicidality because it captures the subjective and functional consequences of distress that symptom scales alone may not fully explain (16, 17). Longitudinal evidence indicates that poorer health-related quality of life is associated with greater suicidal ideation, with depression partially mediating this relationship, thereby suggesting that quality of life is both an outcome of psychopathology and a meaningful component of suicide risk formulations (16). In clinical samples with depressive disorders, lower emotional and physical wellbeing have also been linked to suicidal tendencies, further supporting the inclusion of quality-of-life indicators in contemporary models of suicidal ideation (7, 17).

Recent predictive studies likewise support multivariable and profile-sensitive approaches by showing that suicidal thoughts and behaviors are better identified when multiple biopsychosocial indicators are modeled simultaneously rather than considered in isolation (9).

A second reason for adopting a profile-oriented framework is that the emerging literature continues to distinguish strongly between factors associated with suicidal ideation and those associated with the progression to suicidal behavior (4). In other words, risk factors for having suicidal thoughts are not necessarily identical to the factors that govern the transition from thoughts to attempts, which makes direct assessment of both ideation and behavior methodologically essential (4, 5). This distinction is especially important in studies seeking to map the psychological profile of suicidal ideation, because a design that preserves the ideation–behavior boundary can generate findings that are more precise both conceptually and clinically (5).

Against this background, the present study examines the psychological configuration of suicidal ideation through an integrative framework that combines direct assessment of suicidality with indicators of depressive severity, anxiety, stress, emotion regulation difficulties, coping strategies, and quality of life. This design is aligned with recent calls for multivariable and clinically informative models capable of capturing the heterogeneity of suicidal ideation while also preserving the distinction between suicidal thoughts and suicidal behavior. More specifically, the study is based on the assumption that suicidal ideation is associated not with a single isolated predictor, but with a broader configuration of vulnerabilities and protective resources that together contribute to suicide risk severity.

## 2 The present study

Building on recent advances in suicidology, which emphasize the heterogeneity and multidetermined nature of suicidal ideation, the present study adopts an integrative framework that combines emotional distress, emotion regulation difficulties, coping strategies, and quality of life within a single explanatory model (9, 10). In contrast to approaches that examine predictors in isolation, the present study is based on the assumption that suicidal ideation in depressive disorder reflects a broader psychological configuration shaped by the co-occurrence of multiple vulnerability and protective factors (14, 18). Moreover, consistent with current methodological recommendations, the study differentiates between suicidal ideation and suicidal behavior through the use of a direct suicidality assessment, thereby allowing a more precise examination of the psychological correlates of suicide risk (4, 5). This distinction is particularly important given increasing evidence that the factors associated with suicidal thoughts do not fully overlap with those involved in the progression toward suicidal behavior (4).

Within this framework, the present study pursues three interrelated objectives. First, it examines the associations between suicidal ideation and core dimensions of psychological functioning, including depressive severity, anxiety, stress, emotion regulation difficulties, coping strategies, and quality of life. Based on previous literature, it is expected that suicidal ideation will be positively associated with indicators of emotional distress, emotion dysregulation, and maladaptive coping, and negatively associated with quality of life (6, 7, 13, 19).

Second, the study investigates the predictive contribution of these variables within a multivariable framework. Given the cross-sectional design, the term “predictive” is used here in its statistical sense, referring to the contribution of variables to explaining variation in suicidal ideation within the present sample, and does not imply prospective prediction of future suicidal ideation, suicidal behavior, or suicide risk. More specifically, two complementary hierarchical regression models are used to examine the relative contribution of emotional distress, emotion regulation difficulties, quality of life, and behavioral and cognitive coping strategies to suicidal ideation severity. In line with the literature, it is expected that depressive severity, emotion regulation difficulties, and maladaptive coping strategies will emerge as significant positive contributors, whereas quality of life and more adaptive coping strategies will show negative associations with suicidal ideation (11, 13, 16, 19).

Third, the study examines whether the association between depressive severity and suicidal ideation is indirectly linked to emotion regulation difficulties and reduced quality of life. In this regard, the mediation models were designed to test clinically relevant pathways through which depressive symptom severity may be associated with suicidal ideation through broader difficulties in emotional functioning and diminished subjective wellbeing (11, 12, 16).

Taken together, these aims position the present study within a growing body of research that seeks to refine the understanding of suicidal ideation by integrating direct assessment of suicidality with multidimensional psychological indicators, thereby contributing to more comprehensive models of suicide risk in depressive disorder (1, 9).

## 3 Methods

### 3.1 Study design and setting

This was a cross-sectional observational study conducted in a clinical psychiatric setting in Romania. The study was derived from a broader doctoral research project on suicide risk in depression; however, the present article was restricted to the clinical subsample of patients diagnosed with depressive disorder. Data collection began following ethics approval in April 2018 and continued until February 2019. Participants were recruited from the inpatient unit of the Psychiatry III Clinic, County Emergency Clinical Hospital, Cluj-Napoca. A convenience sampling approach was used, with eligible inpatients enrolled as they became available during the recruitment period.

### 3.2 Participants

The study sample included 172 adults with depressive disorder. Participants were aged between 18 and 59 years (*M* = 41.91, SD = 13.70), and the sex distribution was approximately balanced (84 men, 88 women). Clinical eligibility was established by the treating psychiatrist based on a diagnosis of depressive disorder according to DSM-5 criteria, as documented in the patients' medical records. Only patients experiencing a current depressive episode of moderate or severe intensity, as determined by the treating psychiatrist, were included in the study. Exclusion criteria included depressive episodes with psychotic features, bipolar disorder, other psychotic disorders, current substance use disorders, and cognitive impairment severe enough to interfere with informed consent or questionnaire completion.

### 3.3 Procedure

Patients were enrolled only after providing written informed consent. Confidentiality was ensured by assigning each participant a study code. Participants were informed about the purpose of the study, the voluntary nature of participation, and their right to withdraw at any time without consequences. Questionnaires were administered in paper-and-pencil format, each instrument being preceded by its specific instructions. Completion time for the full assessment battery ranged between 25 and 50 min. The broader research protocol from which the present study was derived was conducted in accordance with the Declaration of Helsinki and was approved by the Ethics Committee of the Iuliu Haţieganu University of Medicine and Pharmacy, Cluj-Napoca (approval no. 191, April 19, 2018). The order of administration was: MADRS, C-SSRS, DASS-21R, CERQ, SACS, DERS-SF, and QOLI. Participants identified through the C-SSRS as being at acute or high suicide risk were immediately evaluated by the treating psychiatrist and managed in accordance with the clinic's established suicide risk management protocol.

### 3.4 Measures

#### 3.4.1 Montgomery–Åsberg depression rating scale, MADRS

MADRS (20–22) is a clinician-rated instrument developed to assess the severity of depressive symptomatology. The scale includes **10 items**, each rated on a **7-point scale** from **0** (no abnormality) to **6** (severe), yielding a total score between **0 and 60**. It focuses primarily on the core affective and cognitive symptoms of depression, including sadness, inner tension, pessimistic thoughts, fatigue, and suicidal thoughts, rather than predominantly somatic symptoms. In the present study, the MADRS was used to assess depressive symptom severity.

#### 3.4.2 Columbia suicide severity rating scale, C-SSRS

The Romanian C-SSRS Baseline/Screening version (14 January 2009) was administered. The C-SSRS (5, 23) is a multidimensional instrument that assesses suicidal ideation and suicidal behavior as distinct constructs. In the present study, suicidal ideation severity was assessed for the past month using the standard 0–5 scale, where 0 indicates no suicidal ideation, 1 indicates a wish to be dead, and 5 indicates active suicidal ideation with a specific plan and intent. Ideation intensity was also assessed according to the C-SSRS framework. Suicidal behavior was coded as an ordinal variable based on the most severe behavioral category endorsed (0 = no suicidal behavior, 1 = preparatory acts or behavior, 2 = aborted attempt, 3 = interrupted attempt, and 4 = actual attempt). Lethality was assessed separately and was not included in the suicidal behavior variable. The C-SSRS also distinguishes suicidal behavior from nonsuicidal self-injurious behavior. In the present sample, internal consistency was good for the suicidal ideation severity component (α = 0.87) and acceptable for suicidal behavior (α = 0.67).

#### 3.4.3 Depression anxiety stress scales, DASS-21R

DASS-21R (24–26) is a self-report measure of current emotional distress comprising three subscales assessing **depression**, **anxiety**, and **stress**. Items are rated on a **4-point scale** from **0** to **3**, with higher scores indicating greater symptom severity. In the present study, the DASS-21R was used to assess the current severity of depressive, anxiety, and stress symptoms and was not used as an eligibility criterion. In the present sample, internal consistency was excellent: **α** **=** **0.97** for Depression, **α** **=** **0.93** for Anxiety, and **α** **=** **0.91** for Stress.

#### 3.4.4 Cognitive emotion regulation questionnaire, CERQ

CERQ (27, 28) is a multidimensional self-report instrument developed to assess **cognitive coping strategies** used after negative or stressful events. Unlike broader coping measures, CERQ targets thoughts rather than overt behavior. The questionnaire includes **36 items** and assesses nine **cognitive strategies**: self-blame, acceptance, rumination, positive refocusing, refocus on planning, positive reappraisal, putting into perspective, catastrophizing, and other-blame. In the present sample„ the internal consistency coefficients were as follows: self-blame **α** **=** **0.73**, acceptance **α** **=** **0.73**, rumination **α** **=** **0.79**, positive refocusing **α** **=** **0.83**, refocus on planning **α** **=** **0.77**, positive reappraisal **α** **=** **0.83**, putting into perspective **α** **=** **0.77**, catastrophizing **α** **=** **0.82**, and other-blame **α** **=** **0.86**.

#### 3.4.5 Strategic approach to coping scale, SACS

SACS (29–31) is a self-report instrument designed to assess **behavioral coping strategies in a social context**. In contrast to cognitive coping measures, SACS focuses on overt behavioral responses to stressful events. The inventory includes nine **subscales**: assertive action, social relating, seeking social support, cautious action, instinctive action, avoidance, indirect action, antisocial action, and aggressive action. In the present sample, internal consistency coefficients were: assertive action **α** **=** **0.67**, social relating **α** **=** **0.80**, seeking social support **α** **=** **0.78**, cautious action **α** **=** **0.60**, instinctive action **α** **=** **0.71**, avoidance **α** **=** **0.78**, indirect action **α** **=** **0.75**, antisocial action **α** **=** **0.82**, and aggressive action **α** **=** **0.78**; the overall internal consistency of the inventory was **α** **=** **0.87**.

#### 3.4.6 Difficulties in emotion regulation scale – short form, DERS-SF

The DERS-SF (32, 33) is a self-report instrument designed to assess difficulties in emotion regulation. It includes **18 items**, rated on a **5-point Likert scale** from **1** to **5**, with three **reverse-scored items**. The scale assesses six **dimensions** of emotion regulation difficulties, with three **items per dimension**: **strategies**, **non-acceptance**, **impulse**, **goals**, **awareness**, and **clarity**. In the present study, the internal consistency coefficients were **α** **=** **0.91** for Strategies, **α** **=** **0.84** for Non-acceptance, **α** **=** **0.96** for Impulse, **α** **=** **0.95** for Goals, **α** **=** **0.90** for Awareness, and **α** **=** **0.83** for Clarity.

#### 3.4.7 Quality of life inventory, QOLI

The QOLI (34, 35) assesses subjective quality of life across 16 life domains, each evaluated in terms of both importance and satisfaction. The instrument was scored according to the procedure specified in the Romanian QOLI manual. For each domain, satisfaction ratings are weighted according to the importance assigned to that domain, and the resulting weighted scores are combined to obtain the overall quality-of-life score, with higher scores indicating better perceived quality of life. In the present study, the **internal consistency coefficient was** **α** **=** **0.92**.

### 3.5 Statistical analysis

All analyses were performed in *JASP 0.98.0*. Suicidal ideation severity, assessed with the C-SSRS, was the primary outcome. Descriptive statistics were computed for all variables, with continuous data reported as means and standard deviations and categorical data as frequencies and percentages. Associations between suicidal ideation and the psychological variables were examined using Spearman's rho correlations. To account for multiple testing in the exploratory correlational analyses, Benjamini–Hochberg false discovery rate correction was applied across the 29 correlations between suicidal ideation severity and the psychological variables. Predictor–predictor correlations were treated as descriptive analyses and were not included in the FDR correction.

Two hierarchical multiple regression models were conducted to examine the relative contribution of the psychological variables to suicidal ideation severity. The first model included depressive severity, emotional distress, emotion regulation difficulties, and quality of life, whereas the second included behavioral coping and cognitive emotion regulation strategies. Mediation analyses with bootstrapping (5,000 samples) were also performed to test the indirect association of depressive severity with suicidal ideation through emotion regulation difficulties and quality of life. The DERS dimensions examined as potential mediators were selected based on their conceptual relevance to suicidal ideation and their significant bivariate associations with suicidal ideation in the present sample, rather than on their significance as independent coefficients in the multivariable regression model. Accordingly, limited access to emotion regulation strategies (DERS strategies) and impulse control difficulties (DERS impulse) were examined in the parallel mediation model. The serial mediation model focused on DERS strategies because limited access to effective regulation strategies provided the clearest conceptual link between depressive severity, impaired emotion regulation, reduced quality of life, and suicidal ideation, allowing a more parsimonious test of the hypothesized sequential pathway. Statistical significance was set at *p* < 0.05.

## 4 Results

Table 1 presents the sociodemographic characteristics of the clinical sample, which comprised 172 participants. The mean age was 41.91 years (SD = 13.70), with ages ranging from 18 to 59 years. The sample was nearly evenly distributed by sex, including 84 men (48.8%) and 88 women (51.2%). Most participants resided in rural areas (62.8%), while 37.2% lived in urban settings. Regarding marital status, equal proportions of participants were unmarried (37.2%) and married (37.2%), whereas 18.6% were divorced and 7.0% were widowed. Educational attainment varied, with high school education being the most common level (46.5%), followed by a bachelor's degree (23.3%), post-secondary education (16.3%), postgraduate education (7.0%), secondary education (4.7%), and primary education (2.3%). In terms of occupational status, almost half of the participants were employed (46.5%), while 23.3% were retired, 11.6% were students, 11.6% reported having no occupation, and 7.0% were unemployed.

**Table 1:** Sociodemographic characteristics of the clinical sample (*N* = 172).

Characteristic	Category / Range	Value, N (%) or M (SD)
Age, M (SD)	Range 18–59	41.91 (13.70)
Sex, *n* (%)	Male	84 (48.8%)
	Female	88 (51.2%)
Area of residence (%)	Rural	108 (62.8%)
	Urban	64 (37.2%)
Marital status (%)	Unmarried	64 (37.2%)
	Married	64 (37.2%)
	Divorced	32 (18.6%)
	Widowed	12 (7.0%)
Education (%)	Primary	4 (2.3%)
	Secondary	8 (4.7%)
	High school	80 (46.5%)
	Post-secondary	28 (16.3%)
	Bachelor's degree	40 (23.3%)
	Postgraduate	12 (7.0%)
Occupational status (%)	Student	20 (11.6%)
	Employed	80 (46.5%)
	Unemployed	12 (7.0%)
	No occupation	20 (11.6%)
	Retired	40 (23.3%)

Results in Table 2 showed that suicidal ideation severity and suicidal behavior were strongly positively associated with each other (ρ = 0.88, *p* < 0.001), indicating that the two suicidality indicators were closely related, although analytically distinct. Both variables showed a similar pattern of associations with depressive severity, emotional distress, and emotion regulation difficulties. Specifically, suicidal ideation severity was positively correlated with MADRS depression (ρ = 0.27, *p* < 0.001), DASS depression (ρ = 0.21, *p* < 0.01), DASS anxiety (ρ = 0.33, *p* < 0.001), and DASS stress (ρ = 0.29, *p* < 0.001). A similar pattern was observed for suicidal behavior. Regarding emotion regulation, suicidal ideation severity was positively associated with limited access to emotion regulation strategies (ρ = 0.37, *p* < 0.001), nonacceptance of emotional responses (ρ = 0.28, *p* < 0.001), impulse control difficulties (ρ = 0.39, *p* < 0.001), and difficulties engaging in goal-directed behavior (ρ = 0.34, *p* < 0.001), whereas no significant associations were found for emotional awareness or emotional clarity. The same pattern of associations was also observed for suicidal behavior. Quality of life was negatively correlated with both suicidal ideation severity and suicidal behavior (ρ = −0.34, *p* < 0.001), indicating that lower perceived quality of life was associated with higher suicidality. In addition, moderate to strong positive intercorrelations were observed among depressive severity, emotional distress, and most dimensions of emotion regulation difficulties, whereas quality of life showed consistent negative associations with distress and emotion dysregulation variables.

**Table 2:** Mean, standard deviation and Spearman correlations among suicidality, depression, distress, emotion regulation difficulties, and quality of life (*N* = 172).

Variables	1	2	3	4	5	6	7	8	9	10	11	12	13	Mean	SD
1. Suicidal ideation severity	–													2.40	0.81
2. Suicidal behavior	0.88^***^	–												1.33	0.60
3. MADRS depression	0.27^***^	0.27^***^	–											31.16	9.79
4. DASS depression	0.21^**^	0.21^**^	0.14	–										13.84	5.48
5. DASS anxiety	0.33^***^	0.33^***^	0.41^***^	0.32^***^	–									10.51	5.48
6. DASS stress	0.29^***^	0.29^***^	0.22^**^	0.59^***^	0.72^***^	–								13.07	5.49
7. DERS strategies	0.37^***^	0.37^***^	0.32^***^	0.42^***^	0.54^***^	0.50^***^	–							10.88	3.66
8. DERS nonacceptance	0.28^***^	0.28^***^	0.35^***^	0.27^***^	0.78^***^	0.48^***^	0.64^***^	–						9.19	3.49
9. DERS impulse	0.39^***^	0.39^***^	0.02	0.45^***^	0.44^***^	0.59^***^	0.61^***^	0.43^***^	–					9.23	4.40
10. DERS goals	0.34^***^	0.34^***^	0.31^***^	0.42^***^	0.51^***^	0.52^***^	0.76^***^	0.61^***^	0.63^***^	–				11.12	3.83
11. DERS awareness	0.14	0.14	−0.05	−0.01	0.07	0.09	0.06	−0.00	−0.06	−0.09	–			8.77	3.10
12. DERS clarity	0.01	0.01	−0.02	0.31^***^	0.39^***^	0.47^***^	0.35^***^	0.46^***^	0.27^***^	0.48^***^	0.12	–		8.05	2.81
13. QOLI total	−0.34^***^	−0.34^***^	−0.03	−0.55^***^	−0.20^**^	−0.31^***^	−0.37^***^	−0.19^*^	−0.38^***^	−0.21^**^	−0.10	−0.01	–	8.14	27.34

Values are Spearman's rho coefficients. ^*^p < 0.05, ^**^p **<** 0.01, ^***^p **<** 0.001.

The mean MADRS score was 31.16 (SD = 9.79), indicating substantial depressive symptom severity and falling within the moderate-to-severe range according to the severity classification used in this study.

Results in Table 3 showed that suicidal ideation severity and suicidal behavior were strongly positively associated with each other (ρ = 0.88, *p* < 0.001). Both variables showed a similar pattern of associations with behavioral coping strategies. Specifically, suicidal ideation severity was positively correlated with indirect action (ρ = 0.18, *p* < 0.05), antisocial action (ρ = 0.32, *p* < 0.001), avoidance (ρ = 0.20, *p* < 0.01), and aggressive action (ρ = 0.17, *p* < 0.05), indicating that higher suicidality was associated with a more maladaptive behavioral coping profile. No significant associations were observed for assertive action, cautious action, social relating, instinctive action, or seeking social support. The same pattern of associations was also observed for suicidal behavior. In addition, strong positive intercorrelations were found among several maladaptive coping dimensions, particularly antisocial action, aggressive action, and instinctive action, suggesting substantial overlap among dysfunctional behavioral coping responses.

**Table 3:** Mean, standard deviation and Spearman correlations among suicidality and behavioral coping strategies (SACS) (*N* = 172).

Variables	1	2	3	4	5	6	7	8	9	10	11	Mean	SD
1. Suicidal ideation severity	–											2.40	0.81
2. Suicidal behavior	0.88^***^	–										1.33	0.60
3. SACS assertive action	−0.06	−0.06	–									28.09	6.65
4. SACS cautious action	0.02	0.02	0.34^***^	–								15.84	4.01
5. SACS indirect action	0.18^*^	0.18^*^	0.47^***^	0.40^***^	–							10.84	4.29
6. SACS social relating	0.00	0.00	0.11	0.50^***^	0.03	–						16.77	4.75
7. SACS instinctive action	0.10	0.10	0.67^***^	0.29^***^	0.62^***^	0.08	–					18.86	5.12
8. SACS antisocial action	0.32^***^	0.32^***^	0.55^***^	0.39^***^	0.82^***^	−0.05	0.71^***^	–				12.93	5.97
9. SACS seeking social support	−0.08	−0.08	0.02	0.41^***^	0.18^*^	0.62^***^	0.03	0.08	–			21.26	6.41
10. SACS avoidance	0.20^**^	0.20^**^	−0.45^***^	0.24^**^	−0.04	0.38^***^	−0.27^***^	−0.03	0.50^***^	–		16.95	5.45
11. SACS aggressive action	0.17^*^	0.17^*^	0.63^***^	0.37^***^	0.71^***^	−0.06	0.77^***^	0.84^***^	−0.03	−0.19^*^	–	12.91	5.23

Values of Spearman's rho coefficients ^**^p < 0.05, ^**^p < 0.01, ^***^p < 0.001

Results in Table 4 showed that suicidal ideation severity and suicidal behavior were strongly positively associated with each other (ρ = 0.88, *p* < 0.001). Both variables showed a similar pattern of associations with cognitive emotion regulation strategies. Specifically, suicidal ideation severity was positively correlated with self-blame (ρ = 0.25, *p* < 0.01), catastrophizing (ρ = 0.19, *p* < 0.05), and especially other-blame (ρ = 0.42, *p* < 0.001). Negative associations were observed for positive reappraisal (ρ = −0.20, *p* < 0.01) and, at the threshold of statistical significance, for refocus on planning (ρ = −0.15, *p* = 0.05). No significant associations were found for acceptance, rumination, positive refocusing, or putting into perspective. The same pattern of associations was also observed for suicidal behavior. In addition, moderate to strong positive intercorrelations were observed among several cognitive emotion regulation strategies, particularly among positive refocusing, refocus on planning, positive reappraisal, and putting into perspective, indicating substantial overlap among the more adaptive cognitive coping dimensions.

**Table 4:** Mean, standard deviation and Spearman correlations among suicidality and cognitive emotion regulation strategies (CERQ) (*N* = 172).

Variables	1	2	3	4	5	6	7	8	9	10	11	Mean	SD
1. Suicidal ideation severity	–											2.40	0.81
2. Suicidal behavior	0.88^***^	–										1.33	0.60
3. CERQ self-blame	0.25^**^	0.25^**^	–									13.00	3.98
4. CERQ acceptance	0.10	0.10	0.28^***^	–								13.19	4.25
5. CERQ rumination	−0.04	−0.04	0.48^***^	0.24^**^	–							13.67	4.21
6. CERQ positive refocusing	0.02	0.02	0.07	0.27^***^	0.31^***^	–						10.33	4.75
7. CERQ refocus on planning	−0.15	−0.15	−0.11	0.09	0.37^***^	0.30^***^	–					14.23	4.07
8. CERQ positive reappraisal	−0.20^**^	−0.20^**^	−0.07	0.19^*^	0.46^***^	0.69^***^	0.61^***^	–				12.19	4.83
9. CERQ putting into perspective	0.03	0.03	−0.08	0.33^***^	0.23^**^	0.61^***^	0.55^***^	0.69^***^	–			11.53	4.49
10. CERQ catastrophizing	0.19^*^	0.19^*^	0.22^**^	0.17^*^	0.21^**^	0.04	0.02	−0.16^*^	0.09	–		12.72	4.54
11. CERQ other-blame	0.42^***^	0.42^***^	0.06	0.30^***^	0.08	0.18^*^	0.03	0.07	0.27^***^	0.47^***^	–	10.58	4.94

Values are Spearman's rho coefficients. ^*^p < 0.05, ^**^p < 0.01, ^***^p < 0.001.

Given the number of correlations examined, a Benjamini–Hochberg false discovery rate correction was applied to the 29 associations between suicidal ideation severity and the psychological variables reported across Tables 2–4. Adjusted *p*-values are presented in Supplementary Table S1. Most of the principal associations described above remained statistically significant after FDR correction, whereas several weaker associations did not survive adjustment and should therefore be interpreted as exploratory.

A hierarchical multiple regression analysis was conducted to examine the contribution of depressive severity, emotional distress, emotion regulation difficulties, and quality of life to suicidal ideation severity (Table 5). In Step 1, which included MADRS depression, DASS depression, DASS anxiety, and DASS stress, the model was statistically significant, *R*^2^ = 0.143, adjusted *R*^2^ = 0.123, *F* = 6.99, *p* < 0.001. Within this step, MADRS depression emerged as the only significant positive predictor (β = 0.302, *p* < 0.001). In Step 2, after adding the dimensions of emotion regulation difficulties, the model remained significant and explained a greater proportion of variance, *R*^2^ = 0.254, adjusted *R*^2^ = 0.207, Δ*R*^2^ = 0.110, *F* = 5.47, *p* < 0.001. In this step, MADRS depression remained a significant positive predictor (β = 0.246, *p* = 0.003), while DERS awareness also emerged as a significant positive predictor (β = 0.160, *p* = 0.032). In Step 3, after including quality of life, the model explained 28.4% of the variance in suicidal ideation, *R*^2^ = 0.284, adjusted *R*^2^ = 0.235, Δ*R*^2^ = 0.031, *F* = 5.78, *p* < 0.001. In the final model, MADRS depression remained a significant positive predictor (β = 0.274, *p* = 0.001), whereas quality of life emerged as a significant negative predictor (β = −0.249, *p* = 0.010), indicating that lower perceived quality of life was associated with higher levels of suicidal ideation.

**Table 5:** Hierarchical regression Model 1: depression, distress, emotion regulation difficulties, and quality of life.

Predictor	Step 1 β [95% CI]	*p*	Step 2 β [95% CI]	*P*	Step 3 β [95% CI]	*p*	Final VIF	Tolerance
MADRS depression	0.302 [0.144, 0.460]	< 0.001	0.246 [0.084, 0.408]	0.003	0.274 [0.113, 0.434]	0.001	1.47	0.678
DASS depression	0.145 [−0.033, 0.322]	0.109	0.077 [−0.096, 0.250]	0.383	−0.060 [−0.259, 0.139]	0.551	2.27	0.441
DASS anxiety	0.118 [−0.109, 0.345]	0.306	−0.052 [−0.339, 0.234]	0.719	0.000 [−0.284, 0.284]	0.998	4.63	0.216
DASS stress	−0.127 [−0.369, 0.114]	0.300	−0.145 [−0.412, 0.123]	0.287	−0.156 [−0.419, 0.107]	0.244	3.96	0.252
DERS strategies	—	—	0.235 [−0.031, 0.500]	0.083	0.114 [−0.163, 0.391]	0.417	4.39	0.228
DERS nonacceptance	—	—	0.096 [−0.151, 0.344]	0.444	0.058 [−0.187, 0.303]	0.639	3.44	0.291
DERS impulse	—	—	0.147 [−0.061, 0.356]	0.165	0.130 [−0.075, 0.335]	0.211	2.41	0.415
DERS goals	—	—	−0.000 [−0.260, 0.260]	0.999	0.065 [−0.195, 0.325]	0.624	3.87	0.258
DERS awareness	—	—	0.160 [0.014, 0.305]	0.032	0.120 [−0.026, 0.266]	0.107	1.23	0.816
DERS clarity	—	—	−0.155 [−0.327, 0.016]	0.076	−0.087 [−0.264, 0.089]	0.328	1.78	0.562
QOLI total	—	—	—	—	−0.249 [−0.436, −0.061]	0.010	2.02	0.496

β, standardized regression coefficient; CI, confidence interval. VIF and tolerance refer to the final model (Step 3). All Model 1 VIF values were < 5. *N* = 172.

Model summary indices are presented in Table 6, indicating a progressive increase in explained variance across steps, with the largest increment following the inclusion of emotion regulation difficulties (Δ*R*^2^ = 0.110), and a smaller but significant contribution of quality of life in the final step (Δ*R*^2^ = 0.031).

**Table 6:** Model summary for hierarchical regression model 1.

Model	*R* ^2^	Adjusted *R*^2^	Δ*R*^2^	*F* (df1, df2)	*p* model	Δ*F* (df1, df2)	*p* change
Step 1	0.143	0.123	0.143	6.99 (4, 167)	< 0.001	6.99 (4, 167)	< 0.001
Step 2	0.254	0.207	0.110	5.47 (10, 161)	< 0.001	3.97 (6, 161)	0.001
Step 3	0.284	0.235	0.031	5.78 (11, 160)	< 0.001	6.85 (1, 160)	0.010

*F*-values include numerator and denominator degrees of freedom. Δ*F* tests the incremental change in explained variance from the preceding step. *N* = 172.

### 4.1 Sensitivity analysis

Given the ordinal nature of C-SSRS suicidal ideation severity, an ordinal logistic regression was conducted as a sensitivity analysis for the final Model 1 (Supplementary Table S2). Higher MADRS depression severity remained significantly associated with increased odds of being in a higher suicidal-ideation category (OR = 1.73 per 1-SD increase, 95% CI 1.10–2.72, *p* = 0.018). The direction of the association for quality of life was consistent with the linear regression model, although it did not reach statistical significance in the ordinal model (OR = 0.59, 95% CI 0.33–1.06, p = 0.077). These findings provide partial support for the robustness of the primary model while also indicating that individual predictor estimates vary according to model specification.

A second hierarchical regression model examined the contribution of behavioral and cognitive coping strategies to suicidal ideation severity (Table 7). In Step 1, which included the SACS dimensions, the model was statistically significant, *R*^2^ = 0.316, adjusted *R*^2^ = 0.278, *F* = 8.33, *p* < 0.001. Significant predictors in this step were assertive action (β = −0.565, *p* < 0.001), indirect action (β = −0.281, *p* = 0.033), social relating (β = 0.378, *p* < 0.001), antisocial action (β = 0.861, *p* < 0.001), and seeking social support (β = −0.324, *p* = 0.001).

**Table 7:** Hierarchical regression Model 2: behavioral and cognitive coping strategies.

Predictor	Step 1 β [95% CI]	*p*	Step 2 β [95% CI]	*P*	Final VIF	Tolerance
SACS assertive action	−0.565 [−0.827, −0.304]	< 0.001	−0.549 [−0.856, −0.243]	0.001	6.35	0.157
SACS cautious action	−0.108 [−0.302, 0.086]	0.272	0.031 [−0.190, 0.252]	0.781	3.31	0.302
SACS indirect action	−0.281 [−0.539, −0.023]	0.033	−0.376 [−0.677, −0.075]	0.015	6.13	0.163
SACS social relating	0.378 [0.169, 0.587]	< 0.001	0.324 [0.091, 0.557]	0.007	3.67	0.272
SACS instinctive action	−0.007 [−0.229, 0.215]	0.952	−0.160 [−0.420, 0.099]	0.224	4.56	0.219
SACS antisocial action	0.861 [0.543, 1.180]	< 0.001	1.024 [0.582, 1.466]	< 0.001	13.21	0.076
SACS seeking social support	−0.324 [−0.519, −0.129]	0.001	−0.428 [−0.625, −0.232]	< 0.001	2.61	0.383
SACS avoidance	−0.073 [−0.305, 0.159]	0.536	0.084 [−0.170, 0.338]	0.514	4.35	0.230
SACS aggressive action	−0.008 [−0.280, 0.265]	0.956	−0.062 [−0.335, 0.211]	0.653	5.05	0.198
CERQ self-blame	—	—	−0.059 [−0.255, 0.137]	0.551	2.59	0.386
CERQ acceptance	—	—	−0.043 [−0.208, 0.123]	0.611	1.86	0.539
CERQ rumination	—	—	0.025 [−0.189, 0.238]	0.820	3.09	0.324
CERQ positive refocusing	—	—	0.053 [−0.179, 0.285]	0.650	3.64	0.275
CERQ refocus on planning	—	—	0.248 [0.025, 0.470]	0.029	3.34	0.299
CERQ positive reappraisal	—	—	−0.417 [−0.733, −0.101]	0.010	6.75	0.148
CERQ putting into perspective	—	—	0.396 [0.168, 0.624]	0.001	3.53	0.284
CERQ catastrophizing	—	—	−0.227 [−0.400, −0.054]	0.010	2.02	0.495
CERQ other-blame	—	—	0.117 [−0.147, 0.381]	0.384	4.71	0.212

β, standardized regression coefficient; CI, confidence interval. VIF and tolerance refer to the final model (Step 2). SACS antisocial action showed VIF = 13.21 and tolerance = 0.076; several additional predictors had VIF > 5. Individual coefficients should therefore be interpreted cautiously. *N* = 172.

In Step 2, after adding the CERQ variables, the model remained statistically significant and explained a greater proportion of variance, *R*^2^ = 0.421, adjusted *R*^2^ = 0.352, Δ*R*^2^ = 0.104, *F* = 6.17, *p* < 0.001. In the final model, significant predictors were assertive action (β = −0.549, *p* = 0.001), indirect action (β = −0.376, *p* = 0.015), social relating (β = 0.324, *p* = 0.007), antisocial action (β = 1.024, *p* < 0.001), seeking social support (β = −0.428, *p* < 0.001), refocus on planning (β = 0.248, *p* = 0.029), positive reappraisal (β = −0.417, *p* = 0.010), putting into perspective (β = 0.396, *p* = 0.001), and catastrophizing (β = −0.227, *p* = 0.010).

Model summary (Table 8) indices indicated a substantial increase in explained variance after the inclusion of cognitive coping strategies (Δ*R*^2^ = 0.104), suggesting that these variables contributed additional explanatory value beyond behavioral coping.

**Table 8:** Model summary for hierarchical regression model 2.

Model	*R* ^2^	Adjusted *R*^2^	Δ*R*^2^	*F* (df1, df2)	*p* model	Δ*F* (df1, df2)	*p* change	Predictors	Cases/ predictor
Step 1	0.316	0.278	0.316	8.33 (9, 162)	< 0.001	8.33 (9, 162)	< 0.001	9	19.1
Step 2	0.421	0.352	0.104	6.17 (18, 153)	< 0.001	3.06 (9, 153)	0.002	18	9.6

Step 2 contains 18 simultaneous predictors, corresponding to 9.6 cases per predictor. ΔF tests the incremental contribution of the CERQ block. N = 172.

As can be seen in Figure 1, results showed that depressive severity had a significant and positive total effect on suicidal ideation (β = 0.27, SE = 0.05, *t* = 5.12, *p* < 0.001). The findings also showed that all simple path coefficients were significant. Primarily, depressive severity was positively associated with emotion regulation difficulties, particularly limited access to emotion regulation strategies (β = 0.32, SE = 0.04, *t* = 7.84, *p* < 0.001) and impulse control difficulties (β = 0.29, SE = 0.05, *t* = 6.21, *p* < 0.001).

**Figure 1:**
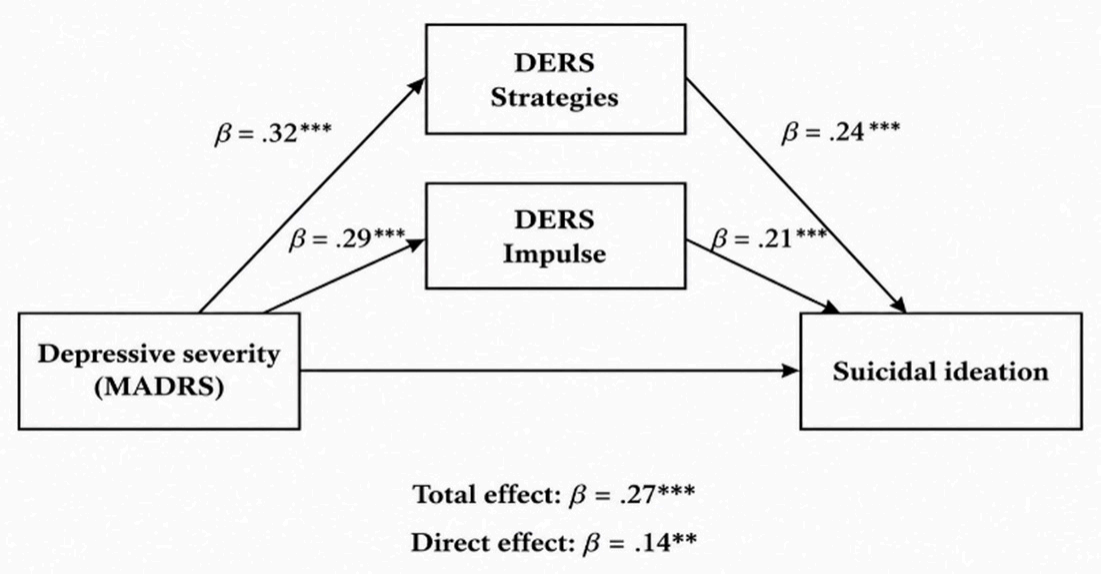
Parallel mediation model examining the indirect effects of depressive severity on suicidal ideation through emotion regulation difficulties. Standardized regression coefficients (β) are presented. *p* < 0.01, *p* < 0.001. DERS, Difficulties in Emotion Regulation Scale; MADRS, Montgomery–Åsberg Depression Rating Scale. ^**^p < 0.01, ^***^p < 0.001.

In turn, emotion regulation difficulties were positively associated with suicidal ideation, with significant effects observed for limited access to emotion regulation strategies (β = 0.24, SE = 0.06, *t* = 3.98, *p* < 0.001) and impulse control difficulties (β =.21, SE = 0.05, *t* = 3.76, *p* < 0.001).

The 95% bias-corrected confidence interval based on 5,000 bootstrap samples indicated that the indirect effects were significant. Specifically, the indirect effect of depressive severity on suicidal ideation through emotion regulation strategies was significant (Effect =.08, SE = 0.02, 95% CI [0.04, 0.13]), and through impulse control difficulties was also significant (Effect = 0.06, SE = 0.02, 95% CI [0.02, 0.10]).

These findings indicate that emotion regulation difficulties partially mediated the association between depressive severity and suicidal ideation, as the confidence intervals did not include zero.

However, the direct effect of depressive severity on suicidal ideation remained significant after including the mediators (β = 0.14, SE = 0.04, *t* = 2.89, *p* = 0.004), suggesting a pattern of partial mediation.

Overall, the results support the hypothesis that deficits in emotion regulation represent a key psychological mechanism through which depressive severity contributes to suicidal ideation.

As can be seen in Figure 2, results showed that depressive severity had a significant and positive total effect on suicidal ideation (β = 0.27, SE = 0.05, *t* = 5.12, *p* < 0.001).

**Figure 2:**
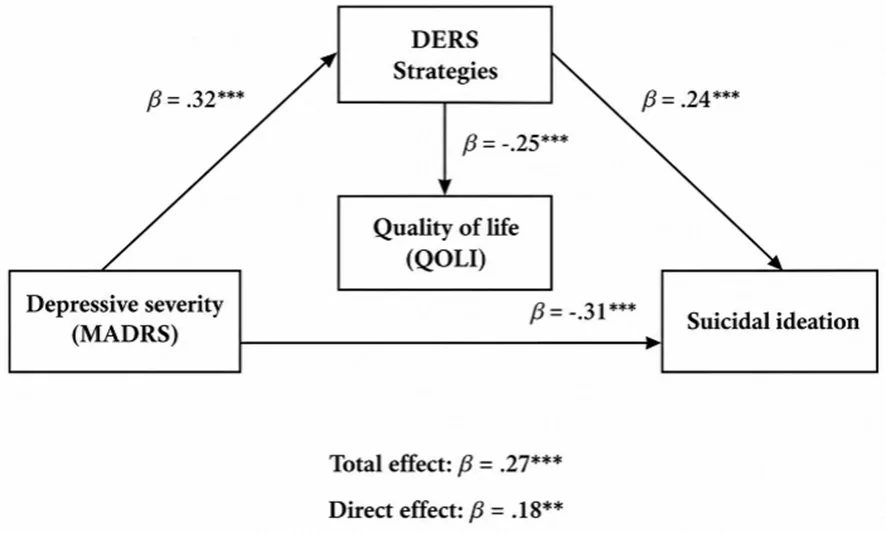
Serial mediation model examining the indirect association between depressive severity and suicidal ideation through emotion regulation difficulties and quality of life. Standardized regression coefficients (β) are presented. ^*^p < 0.01, ^***^p < 0.001. DERS, Difficulties in Emotion Regulation Scale; MADRS, Montgomery–Åsberg Depression Rating Scale; QOLI, quality of life inventory.

The findings also indicated that all simple path coefficients were significant. Specifically, depressive severity was positively associated with emotion regulation difficulties, particularly limited access to emotion regulation strategies (β = 0.32, SE = 0.04, *t* = 7.84, *p* < 0.001).

In turn, emotion regulation difficulties were negatively associated with quality of life (β = −0.25, SE = 0.05, *t* = −4.96, *p* < 0.001), indicating that greater emotion dysregulation was related to poorer perceived quality of life.

Quality of life was further negatively associated with suicidal ideation (β = −0.31, SE = 0.06, *t* = −5.17, *p* < 0.001), suggesting that lower quality of life was linked to higher levels of suicidal ideation.

Additionally, emotion regulation difficulties remained directly associated with suicidal ideation (β = 0.24, SE = 0.06, *t* = 3.98, *p* < 0.001), while depressive severity also showed a direct effect on suicidal ideation (β = 0.18, SE = 0.05, *t* = 3.21, *p* = 0.002).

The 95% bias-corrected confidence interval based on 5,000 bootstrap samples indicated that the indirect effects were significant. The indirect effect of depressive severity on suicidal ideation through emotion regulation difficulties was significant (Effect =.08, SE = 0.02, 95% CI [0.04, 0.13]).

Importantly, the serial indirect effect of depressive severity on suicidal ideation through emotion regulation difficulties and quality of life was also significant (Effect = 0.05, SE = 0.02, 95% CI [0.02, 0.09]), indicating a sequential mediation pathway.

These findings suggest that depressive severity contributes to suicidal ideation both directly and indirectly through increased emotion regulation difficulties and decreased quality of life. The persistence of the direct effect indicates a pattern of partial serial mediation.

## 5 Discussions

The present study examined the psychological configuration of suicidal ideation in adults with depressive disorder using an integrative framework that combined emotional distress, emotion regulation difficulties, coping strategies, and quality of life. Overall, the findings support the view that suicidal ideation is associated with a multidimensional pattern of psychological vulnerabilities, rather than with a single isolated factor, and are consistent with recent models emphasizing the complexity and heterogeneity of suicide risk (1, 9).

At the bivariate level, suicidal ideation was positively associated with depressive severity, anxiety, stress, and multiple dimensions of emotion regulation difficulties, and negatively associated with quality of life. These findings are consistent with previous research indicating that emotional distress and impaired emotion regulation are central correlates of suicidal thinking in depression (6, 13, 16). The strong association between suicidal ideation and suicidal behavior further supports the related but distinct nature of these constructs, in line with contemporary suicidology frameworks (4, 5).

The hierarchical regression analyses provided additional insight into the relative contribution of these variables. In the first model, depressive severity emerged as the most robust predictor of suicidal ideation, while quality of life showed a significant negative association in the final step. These findings suggest that, beyond symptom severity, reduced subjective wellbeing represents an important correlate of suicidal ideation, consistent with previous studies highlighting the protective role of quality of life in depression (12, 16). Emotion regulation difficulties contributed to the model, although their effects were less stable when considered alongside other variables, suggesting potential overlap with broader dimensions of distress. Notably, DERS Awareness was not significantly associated with suicidal ideation at the bivariate level but emerged as a significant coefficient when the emotion regulation dimensions were entered simultaneously in the regression model. Given the shared variance among DERS dimensions, this pattern may reflect a conditional or suppression-related effect within the multivariable model rather than a stronger direct association between Awareness and suicidal ideation; accordingly, Awareness was not selected for the mediation analyses.

The second regression model indicated that behavioral and cognitive coping strategies contributed additional explanatory value to suicidal ideation severity. At the bivariate level, suicidal ideation was positively associated with several maladaptive coping strategies, including antisocial action, aggressive action, self-blame, and other-blame, while positive reappraisal showed a negative association. However, these bivariate associations did not uniformly translate into independent contributions in the multivariable model. In particular, self-blame, other-blame, and aggressive action were not significant in the final regression model. Given the substantial intercorrelations and multicollinearity among coping dimensions, individual regression coefficients should be interpreted cautiously; accordingly, Model 2 is more appropriately interpreted in terms of the overall and incremental explanatory contribution of the behavioral and cognitive coping blocks rather than as evidence of stable independent effects of individual coping strategies.

The mediation analyses further supported the role of emotion regulation difficulties as an important pathway linking depressive severity and suicidal ideation. Specifically, limited access to emotion regulation strategies and impulse control difficulties were associated with higher levels of suicidal ideation and statistically accounted for part of the association between depressive severity and suicidal ideation. In addition, the serial mediation model suggested that this association was also linked to reduced quality of life, indicating that emotion dysregulation and diminished subjective wellbeing may operate together within a broader psychological pathway. Importantly, the direct effect of depressive severity remained significant, indicating partial mediation and suggesting that multiple mechanisms are likely involved. Given the cross-sectional design, these findings should be interpreted as evidence of indirect associations rather than causal pathways.

From a clinical perspective, the findings highlight several relevant targets for assessment and intervention (36). First, they underscore the importance of evaluating not only depressive severity but also emotion regulation difficulties and perceived quality of life when assessing suicide risk. Second, the results suggest that interventions aimed at improving emotion regulation skills and enhancing quality of life may be particularly relevant for reducing suicidal ideation in patients with depressive disorder. Third, the identification of maladaptive coping patterns, such as antisocial or self-blaming responses, may inform more individualized treatment approaches that target specific dysfunctional strategies.

From a suicide prevention and public health perspective, the present findings support the value of broadening risk assessment beyond the identification of depressive symptoms alone. The associations of suicidal ideation with emotion regulation difficulties, maladaptive coping patterns, and reduced quality of life suggest that potentially modifiable psychological and functional factors may provide clinically relevant information alongside conventional assessments of symptom severity. Incorporating brief measures of these domains into routine mental health assessment may help identify individuals with depression who experience a broader constellation of vulnerabilities associated with suicidal ideation and may inform more individualized preventive interventions. At the service level, the findings also support integrated suicide prevention approaches that combine systematic assessment of suicidal ideation with interventions targeting emotion regulation, adaptive coping, and psychosocial functioning. Such an approach is consistent with a public health perspective in which suicide prevention extends beyond the management of psychiatric symptoms to include modifiable factors that influence individuals' capacity to manage distress and maintain functioning and quality of life. Given the cross-sectional nature of the present study, however, the utility of these factors for prospective risk stratification and prevention should be established in longitudinal studies before their incorporation into predictive screening models.

Several limitations should be considered when interpreting the findings. The cross-sectional design precludes any conclusions regarding temporal or causal relationships among the variables. Also, the study was conducted in a clinical sample from a single setting, which may limit the generalizability of the findings. The reliance on self-report measures for several constructs may introduce shared method variance and response biases. The inclusion of multiple correlated predictors in the regression models raises the possibility of multicollinearity and suppression effects, which may influence the stability and interpretation of individual coefficients.

The second hierarchical regression model should be considered exploratory given its complexity relative to the available sample size. Its final step included 18 simultaneous predictors, corresponding to 9.6 cases per predictor. In addition, multicollinearity diagnostics identified substantial overlap among several coping dimensions, with VIF values exceeding 5 for multiple predictors and reaching 13.21 for SACS antisocial action. The combination of a relatively low case-to-predictor ratio and multicollinearity increases the risk of coefficient instability, suppression effects, and overfitting. Consequently, individual coefficients from Model 2 should not be interpreted as stable independent effects; greater emphasis should be placed on the overall and incremental contribution of the behavioral and cognitive coping blocks. Replication in larger samples using more parsimonious, cross-validated, dimension-reduction, or penalized regression approaches is warranted.

Additionally, the MADRS total score includes an item assessing suicidal thoughts (item 10), which introduces some conceptual overlap with the C-SSRS suicidal ideation outcome. Consequently, the association between depressive severity and suicidal ideation may have been partly inflated by criterion contamination. Although the total MADRS score was retained to preserve the instrument's standard scoring and the assessment approach specified in the original study protocol, this overlap should be considered when interpreting the findings. Future studies may examine the robustness of this association using depression severity scores that exclude the suicidality item.

The primary regression and mediation analyses treated C-SSRS suicidal ideation severity as an approximately continuous outcome, although the measure is ordinal. This analytic choice may affect coefficient estimates and should be considered when interpreting the findings. An ordinal logistic regression sensitivity analysis supported the association between depressive severity and higher suicidal-ideation severity, although not all individual predictors from the linear model remained statistically significant, indicating some sensitivity to model specification.

The dataset did not include sufficiently standardized information on psychotropic medication, treatment phase, psychiatric comorbidities, previous suicide attempts, or family history of suicide to permit their inclusion as covariates in the present analyses. These factors are clinically relevant correlates of suicidal risk and may have acted as unmeasured confounders. Consequently, the regression models should be interpreted as explanatory models examining selected psychological correlates of suicidal ideation rather than as comprehensive clinical risk-prediction models. Future studies should integrate these psychological dimensions with detailed treatment, diagnostic, and suicide-history variables.

Finally, although the study adopted an integrative framework, it did not employ person-centered analytic approaches, which may further clarify distinct risk profiles in future research.

An additional limitation concerns the time elapsed since data collection, which was conducted in 2018–2019. Although no substantial organizational changes have occurred in Romania in the psychiatric service from which the sample was recruited, the broader mental-health context has evolved, particularly following the COVID-19 pandemic. Global evidence indicates a substantial pandemic-related increase in the burden of depressive and anxiety disorders (37), while findings regarding suicidal outcomes are more heterogeneous. Meta-analytic evidence suggests increases in suicidal ideation and suicide attempts during the pandemic, whereas population suicide mortality did not show a corresponding overall increase (38, 39). Studies conducted in Romania similarly documented a considerable mental-health burden during the pandemic, although national and regional data do not indicate a uniform increase in suicide mortality (40, 41). Accordingly, the present findings should be understood as reflecting a pre-pandemic clinical cohort, and their generalizability to contemporary patients with depressive disorders warrants confirmation in more recent samples.

Future studies may benefit from longitudinal designs that allow for the examination of temporal dynamics and from the use of person-centered methods to identify distinct subgroups of individuals at risk. In addition, incorporating objective or clinician-rated measures alongside self-report instruments may further strengthen the validity of the findings.

## 6 Conclusions

Suicidal ideation in depressive disorder appears to be embedded in a broader psychological configuration marked by depressive severity, emotion regulation difficulties, maladaptive coping, and reduced quality of life. The present findings suggest that suicide risk in depression is better understood through an integrative framework that goes beyond symptom severity alone and incorporates the way individuals regulate distress, cope with adversity, and evaluate their overall wellbeing.

Particularly important is the finding that emotion regulation difficulties and quality of life were meaningfully linked to the association between depressive severity and suicidal ideation, supporting their relevance as clinically significant correlates of suicide risk. These results reinforce the need for suicide risk assessment models that do not focus exclusively on depressive symptoms, but also consider broader mechanisms of emotional and functional vulnerability.

From a clinical perspective, the study highlights emotion regulation, coping processes, and quality of life as relevant targets for intervention in patients with depressive disorder presenting suicidal ideation. Overall, the findings contribute to a more nuanced and clinically useful understanding of suicidal ideation and support the development of more comprehensive approaches to suicide prevention in depression.

## Data Availability

The raw data supporting the conclusions of this article will be made available by the authors, without undue reservation.
