# Supplementary material for: The psychological configuration of suicidal ideation in depressive disorder: an integrative and predictive approach
Source: Front Public Health. 2026 Oct 9;14:1958636. doi: 10.3389/fpubh.2026.1958636 (PMC13690632; doi:10.3389/fpubh.2026.1958636)
Supplement: Supplementary file 1 [file Table_1.docx]

**Supplementary Table S1. Benjamini–Hochberg FDR correction for correlations with suicidal ideation severity**

| **Variable** | **Spearman ρ** | **Unadjusted p** | **BH-FDR adjusted p** | **Significant after FDR** |
| --- | --- | --- | --- | --- |
| MADRS depression | 0.272 | <.001 | .001 | Yes |
| DASS depression | 0.213 | .005 | .012 | Yes |
| DASS anxiety | 0.328 | <.001 | <.001 | Yes |
| DASS stress | 0.287 | <.001 | <.001 | Yes |
| DERS strategies | 0.370 | <.001 | <.001 | Yes |
| DERS nonacceptance | 0.284 | <.001 | .001 | Yes |
| DERS impulse | 0.386 | <.001 | <.001 | Yes |
| DERS goals | 0.339 | <.001 | <.001 | Yes |
| DERS awareness | 0.138 | .071 | .109 | No |
| DERS clarity | 0.015 | .850 | .880 | No |
| QOLI total | -0.339 | <.001 | <.001 | Yes |
| SACS assertive action | -0.057 | .461 | .581 | No |
| SACS cautious action | 0.019 | .804 | .880 | No |
| SACS indirect action | 0.183 | .016 | .029 | Yes |
| SACS social relating | 0.003 | .964 | .964 | No |
| SACS instinctive action | 0.099 | .196 | .284 | No |
| SACS antisocial action | 0.320 | <.001 | <.001 | Yes |
| SACS seeking social support | -0.077 | .315 | .415 | No |
| SACS avoidance | 0.200 | .009 | .018 | Yes |
| SACS aggressive action | 0.174 | .023 | .038 | Yes |
| CERQ self-blame | 0.247 | .001 | .003 | Yes |
| CERQ acceptance | 0.095 | .213 | .294 | No |
| CERQ rumination | -0.045 | .562 | .679 | No |
| CERQ positive refocusing | 0.018 | .819 | .880 | No |
| CERQ refocus on planning | -0.150 | .050 | .081 | No |
| CERQ positive reappraisal | -0.200 | .009 | .018 | Yes |
| CERQ putting into perspective | 0.028 | .717 | .831 | No |
| CERQ catastrophizing | 0.185 | .015 | .029 | Yes |
| CERQ other-blame | 0.424 | <.001 | <.001 | Yes |

Note. Benjamini–Hochberg correction was applied across the 29 correlations between suicidal ideation severity and the psychological variables reported across Tables 2–4. Predictor–predictor intercorrelations were treated as descriptive diagnostics. N = 172.

**Supplementary Table S2. Ordinal logistic regression sensitivity analysis for the final Model 1**

| **Predictor** | **b** | **SE** | **p** | **OR per 1 SD** | **95% CI for OR** |
| --- | --- | --- | --- | --- | --- |
| MADRS depression | 0.548 | 0.231 | .018 | 1.73 | 1.10–2.72 |
| DASS depression | 0.080 | 0.347 | .818 | 1.08 | 0.55–2.14 |
| DASS anxiety | 0.430 | 0.480 | .370 | 1.54 | 0.60–3.93 |
| DASS stress | -0.305 | 0.451 | .498 | 0.74 | 0.30–1.78 |
| DERS strategies | -0.039 | 0.415 | .925 | 0.96 | 0.43–2.17 |
| DERS nonacceptance | -0.172 | 0.380 | .651 | 0.84 | 0.40–1.77 |
| DERS impulse | 0.420 | 0.311 | .176 | 1.52 | 0.83–2.80 |
| DERS goals | 0.896 | 0.448 | .046 | 2.45 | 1.02–5.90 |
| DERS awareness | 0.282 | 0.219 | .198 | 1.33 | 0.86–2.04 |
| DERS clarity | -0.462 | 0.299 | .122 | 0.63 | 0.35–1.13 |
| QOLI total | -0.524 | 0.297 | .077 | 0.59 | 0.33–1.06 |

Note. C-SSRS suicidal ideation severity was modeled as an ordinal outcome using the three observed levels in the clinical sample (2, 3, and 5). Predictors were standardized; OR represents the change in odds of a higher suicidal-ideation category per 1-SD increase. N = 172
